# Cutaneous *Malassezia:* Commensal, Pathogen, or Protector?

**DOI:** 10.3389/fcimb.2020.614446

**Published:** 2021-01-26

**Authors:** Shree Harsha Vijaya Chandra, Ramasamy Srinivas, Thomas L. Dawson, John E. Common

**Affiliations:** ^1^ Skin Research Institute of Singapore, Agency for Science, Technology and Research, Singapore, Singapore; ^2^ Department of Drug Discovery, College of Pharmacy, Medical University of South Carolina, Charleston, SC, United States

**Keywords:** *Malassezia*, commensal, pathogen, mutualist, multikingdom, immunity, skin, host and disease

## Abstract

The skin microbial community is a multifunctional ecosystem aiding prevention of infections from transient pathogens, maintenance of host immune homeostasis, and skin health. A better understanding of the complex milieu of microbe-microbe and host-microbe interactions will be required to define the ecosystem’s optimal function and enable rational design of microbiome targeted interventions. *Malassezia*, a fungal genus currently comprising 18 species and numerous functionally distinct strains, are lipid-dependent basidiomycetous yeasts and integral components of the skin microbiome. The high proportion of *Malassezia* in the skin microbiome makes understanding their role in healthy and diseased skin crucial to development of functional skin health knowledge and understanding of normal, healthy skin homeostasis. Over the last decade, new tools for *Malassezia* culture, detection, and genetic manipulation have revealed not only the ubiquity of *Malassezia* on skin but new pathogenic roles in seborrheic dermatitis, psoriasis, Crohn’s disease, and pancreatic ductal carcinoma. Application of these tools continues to peel back the layers of *Malassezia*/skin interactions, including clear examples of pathogenicity, commensalism, and potential protective or beneficial activities creating mutualism. Our increased understanding of host- and microbe-specific interactions should lead to identification of key factors that maintain skin in a state of healthy mutualism or, in turn, initiate pathogenic changes. These approaches are leading toward development of new therapeutic targets and treatment options. This review discusses recent developments that have expanded our understanding of *Malassezia*’s role in the skin microbiome, with a focus on its multiple roles in health and disease as commensal, pathogen, and protector.

## *Malassezia*: A Major Component of the Skin Microbiome

Human skin serves as our protective physical barrier, but also consists of a complex micro-environmental ecosystem. The skin surface micro-environment is colonized by a wide range of microorganisms, including bacteria, archaea, viruses, and fungi, collectively referred to as the skin microbiome. While initially considered as a 2 m^2^ flat surface representing a smaller and less influential niche than gut or lung, when one considers skin’s 3-dimensional topography it becomes an estimated 30 m^2^, similar in surface area to gut or lung. This, coupled with access to viable epidermis in deeper invaginations (such as the follicle infundibulum), the skin becomes an important and relevant microbial niche (Gallo, 2017). The complex ecosystem in any individual is governed by the skin’s divergent niches, ranging from dry (heel, volar forearm) to moist (antecubital fossa, axilla) to dry and oily (face, upper back) to moist and oily (scalp), driving high microbial variability between niches. While there remains microbial variability between individuals, it is relatively low compared to that between different body site niches (Grice et al., 2009; Oh et al., 2014). Multikingdom DNA sequencing has revealed the skin microbiome has notably higher viral and fungal representation when compared to gut (Arumugam et al., 2011; Oh et al., 2014). The skin microbiome is also temporally stable over long periods regardless of the environmental perturbations experienced in daily life (Oh et al., 2016). Factors such as short term washing with common (non-antimicrobial) hygiene products does not disrupt the skin commensal microbial diversity, but can help displace opportunistic pathogenic colonizers (Two et al., 2016). The eukaryotic component of skin microbiome is dominated by *Malassezia* (Findley et al., 2013; Oh et al., 2014; Jo et al., 2016; Byrd et al., 2018), which are found in highest abundance on sebaceous sites including scalp, face, chest, and upper back, and in lower abundance on trunk and arms. Feet are the exception with lower *Malassezia* content and higher overall fungal diversity (**Table 1**) (Findley et al., 2013). Interestingly, the diversity of commensal skin fungi can also vary with geography and possibly ethnicity in healthy individuals (Leong et al., 2019).

**Table 1:** Summary of currently described *Malassezia* species present in human body sites associated with health and/or disease.

S/N	*Malassezia* Species	Group (**Figure 1**)	Body site^†^ (Human)	Health/Disease^‡^	References
**1**	*M. furfur*	A	D, S, SC, Sy	AD, D, SD, PV, SI, F	(Simmons and Gueho, 1990; Boekhout T et al., 2010; Cabanes, 2014; Iatta et al., 2014)
**2**	*M. obtusa*	A	D, S	AD, D, SD	(Boekhout T et al., 2010; Cabanes, 2014; Prohic et al., 2016)
**3**	*M. japonica*	A	D, S	AD	(Sugita et al., 2003)
**4**	*M. yamatoensis*	A	D, S	AD, SD	(Sugita et al., 2004; Boekhout T et al., 2010)
**5**	*M. sympodialis*	B	D, M, S	D, SD, AD, PV, F	(Gupta et al., 2004; Boekhout T et al., 2010; Aguirre et al., 2015; Patron, 2016)
**6**	*M. dermatis*	B	D, S	AD	(Sugita et al., 2002; Boekhout T et al., 2010; Guého-Kellermann and Begerow, 2010)
**7**	*M. restricta* (all humans)	B	D, M, S, F	D, SD, AD, P	(Nakabayashi et al., 2000; Batra et al., 2005; Boekhout T et al., 2010; Guého-Kellermann and Begerow, 2010)
**8**	*M. globosa* (all humans)	B	D, M, S, F	D, SD, AD, PV, P	(Gupta et al., 2004; Boekhout T et al., 2010; Cabanes, 2014)
**9**	*M. pachydermatis* (normally animal, not human)	B	Sy	SI	(Boekhout T et al., 2010; Chow et al., 2020)
**10**	*M. arunalokei*	B	S, ear	D, SD	(Honnavar et al., 2016)
**11**	*M. slooffiae* (rare)	C	D, S	SD	(Boekhout T et al., 2010)

Non-human associated species: M. brasiliensis (Parrot-Group A); M. psittaci (Parrot-Group A); M. equina (Horse, Cow); M. nana (Cat, cow, Dog-Group B); M. caprae (Goat, Horse-Group B); M. vespertilionis (Bat-Group C); M. cuniculi (Rabbit-Group C). ^†^Body Site: SC-Scalp; F-foot; Sy- systemic (blood, urine); S-sebaceous; M-moist; D-dry. ^‡^Health/Disease: AD-Atopic dermatitis, PV-Pityriasis Versicolor; D-Dandruff; SD-seborrheic dermatitis; P-psoriasis; F-folliculitis; SI-systemic infections.

Metagenomics defines the genetic information from a single microbe as one functional unit, a set of genes, and is used as such to determine the presence of individual microorganisms and predict abundance. However, the relative number of genomes provides an estimate of abundance independent of the cellular size, or interactive biomass, which differs considerably between organisms. For metagenomic ecological analyses, it is important to consider the potential biomass of each microbial community member, as biomass is how bacteria, fungi, or viruses interact with the host as a functional unit. Using conservative estimates of cell size, *Malassezia* have 200–500 times the cellular biomass per genome relative to *Staphylococcus epidermidis*. Hence it would be reasonable and likely meaningful to consider biomass availability during interaction with the host (Ramasamy et al., 2019). Doing so promotes the fungal biomass component of sebaceous skin to at least an equal footing with bacteria.

**Figure 1:**
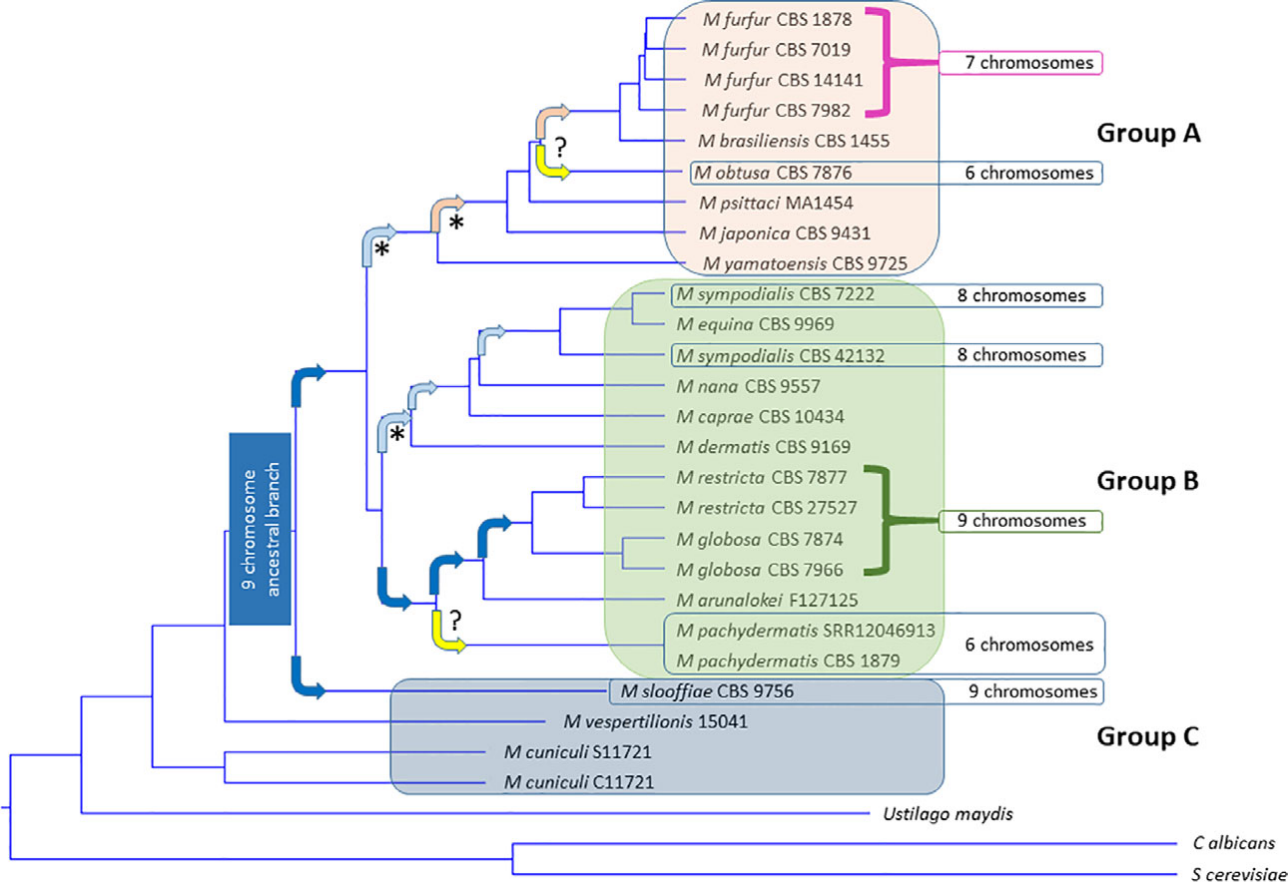
Phylogenetic tree of all 18 currently accepted *Malassezia* species. *Malassezia* can be subdivided into subgroups A, B, and C based on Wu et al. PLoS Genetics 2015. Tree constructed from most current NCBI LSU data (**Table 1**) using the MAFFT method to generate multi-align sequence (MSA), UPGMA (unweighted pair group method with arithmetic mean) for clustering, phylogenetic tree viewed using Newick viewer. MAFFT v6.864 is a multiple alignment program for amino acid or nucleotide sequences (Katoh et al., 2005). (http://www.trex.uqam.ca/index.php?action=mafft and http://mafft.cbrc.jp/alignment/software/). Known species of inhabitation and activities can be found in **Table 1**. Arrows indicate hypothesized chromosome losses associated with development of new species (Sankaranarayanan et al., 2020). Dark blue box = nine chromosome ancestral lineage. Dark blue arrows, nine chromosome lineages. Light blue arrow predicted centromere loss to eight chromosomes. Pink arrow predicted centromere loss to seven chromosomes. Orange arrow unknown chromosome loss to six chromosomes. Yellow unknown event ()? resulting in loss to six chromosomes. (* = documented by centromere loss) (*M. pachydermatis* chromosome number postulated from complete genome assemblies, *M. obtusa* hypothesized based on PGFE karyotype) (Kiuchi et al., 1992; Boekhout et al., 1998; Kim et al., 2018).

Molecular phylogenetic and genomic studies have shown *Malassezia* belong to Basidiomycota, *Ustilagomycotina* and class *Malasseziomycetes*. *Malassezia* have undergone an unfortunate and complex series of nomenclature changes which have clouded their research history. Originally discovered by Malassez and Sabaouraud in the late 19^th^ century, their resistance to cultivation led to the conclusion that there was one species, named *Malassezia* (Malassez, 1874; Sabouraud, 1897). In the 1950’s there were three identified species, renamed as *Pityrosporum* (which is still found in some textbooks): *P. ovale*, named for their oval shape; *P. orbiculare*, for their round shape; and *P*. *pachydermatis*, as the species found on animal as opposed to human skin (Rhoda, 1939). Other nomenclature included *M. furfur* serovars (A, B or C) now re-classified as A-*M. furfur*, B-*M. globosa* and C-*M. restricta* (Cunningham et al., 1990; Ashbee et al., 1993; Batra et al., 2005). Today the *Malassezia* genus is diverse and comprises 18 species, with numerous functionally distinct strains (**Figure 1**) (Theelen et al., 2019). *Malassezia* have haploid genomes of 8–9 Mb, among the smallest for free-living fungi. They have evolved genetic content enriched for genes specific to their environment, encoding lipases, phospholipases and acid sphingomyelinases for utilization of lipids, and proteases for utilization of proteins (Poh et al., 2020). They lack a fatty acid synthase and δ-9 desaturase, likely due to their habitation on oil rich sebaceous skin, and hence have an evolutionary inability to synthesize lipids as part of their adaptation to life on skin (Saunders et al., 2012; White et al., 2014; Wu et al., 2015). The most closely related and well-established fungus is the plant pathogen *Ustilago maydis*, which also targets their plant host for degradation, but *via* secretion of enzymes to break down proteins, pectin, and wax common to plant surfaces. Interestingly, while closely phylogenetically related to *Ustilago*, *Malassezia* secrete an enzyme armada much more similar to the distantly related *Candida albicans*, an example of niche-specific evolution (Xu et al., 2007).

The *Malassezia* clade can be subdivided into three major groups, as seen in **Figure 1** and **Table 1**. Group A are represented as *M. furfur*-like, are more robust in culture, less frequent inhabitants of human skin and more often linked to skin or septic disease. Group B are more common on healthy human skin, with *M. restricta* and *M. globosa* by far the most common and found on the skin of all humans, followed by *M. sympodialis*, then distantly by the other Group B members. The Group B exception is *M. pachydermatis*, which can cause human septic infections but is only normally found on animal skin. **Figure 1** also reveals pathways of chromosomal rearrangements resulting from centromere loss of function (Sankaranarayanan et al., 2020). A nine-chromosome ancestral lineage is hypothesized, which carried through to most of Group B. One centromere was lost to generate the eight-chromosome *M. sympodialis* group, and then another to generate the seven-chromosome Group C. *M. pachydermatis* and *M. obtusa* remain six-chromosome outliers. A series of horizontal gene transfers have been defined and further cloud the phylogeny (Ianiri et al., 2020). For example, another recent LSU tree, while very similar implies different grouping and relationships (Ianiri and Heitman, 2020). These findings highlight that while much has been learned about *Malassezia* phylogeny there is still a long way to go to understanding this unusual clade. Unfortunately, many *Malassezia* species do not have complete genomes, so further long read sequencing and assembly of chromosome-level genomes will be necessary to further refine *Malassezia* evolution. The lack of capability to cultivate many *Malassezia* species and strains has been an impediment to genome sequencing and characterization of gene function. Developing new culture conditions to allow cultivation of new species and continuation of the consortium/repository based exchange of genome information will advance understanding about *Malassezia*. The genomic relatedness of known and sequenced *Malassezia* spp. is useful for future species genome assembly and gene assignment, but unfortunately a large number of *Malassezia* genes fall into the category of unknown function and exist as families of similar gene structure. The reasons for duplication or multiplication of genes in *Malassezia* in the perspective of evolution could be addressed by genome evolution studies (Sankaranarayanan et al., 2020). The availability of more complete *Malassezia* genomes will further the understanding of unknown gene function, identify potential pathways to harness unidentified roles in host-commensal or host-pathogen interactions.

Historically, Malassezia genetic engineering has been extremely challenging. However, it has now been accomplished *via* agrobacterium-mediated transformation (Ianiri et al., 2016; Celis et al., 2017; Ianiri and Heitman, 2020). For example, gene deletion of a bacterially derived flavohemoglobin gene found in all *Malassezia* spp. detoxifies skin generated nitric oxide and is involved in *Malassezia*/Host interaction (Ianiri et al., 2020). Other gene deletion studies have shown that the multidrug transported PDR10 is involved in Malassezia furfur antimicrobial resistance (Ianiri et al., 2019). Finally, insertion of marker and tracking genes into Malassezia for use in *in vitro*, *ex vivo*, and *in vivo* models should assist in more detailed investigation of *Malassezia*/Host interactions (Goh et al., 2020). Improved gene and genome information will assist with further identification of novel proteins that trigger skin disease, inflammation, antifungal response, immunological response of host and mechanistic functions in clinical perspective.

Microbial community and host interactions have multiple effects: broadly classified as commensal, pathogenic, or mutualistic (Schommer and Gallo, 2013). Commensalism is an active relationship between individuals of two species in which one species obtains benefits from the other without benefiting the latter. In a commensal paradigm *Malassezia* obtains the benefit of a food source while causing no effect to the human host. Pathogenicity is a relationship where one member is harmed, in this situation with *Malassezia* activity resulting in direct host damage through specific secreted virulence factors or toxins that negatively affect the host; or indirectly through induction of a damaging host response. In fungal infection, it is necessary to have a functional definition of virulence and pathogenicity, termed a “Damage Response Framework (DRF). In the DRF, a “causal” microorganism may manifest disease directly through products or antigens, or indirectly *via* induction of a harmful host response. In the DRF a pathogen is defined as eliciting a functional change from a commensal to a pathogenic state (Casadevall and Pirofski, 1999; Casadevall and Pirofski, 2003; Casadevall and Pirofski, 2018). Mutualism is classed as an active relationship where both species benefit. For *Malassezia*, they may not only survive on our skin but also may provide protection from contextually pathogenic microbes such as *S. aureus* (Li et al., 2018). Many acute skin infections have underlying microbial contributions which are improved by antimicrobial treatment (Golan, 2019). However, due to the frequency of unknown individual susceptibilities, it is often challenging to successfully satisfy Koch’s postulates to prove causality by individual microbial components (Koch, 1893; Grice and Dawson, 2017). It is therefore important to delineate the context of microbial interactions with skin disease outcomes. Functional interactions can be scenarios where (i) the skin microbiota is a direct cause, (ii) the skin microbiota is altered by changes in the skin and hence generate a deleterious host response, exacerbating the situation, or (iii) where the microbiota is uninvolved. Differentiating these functional interactions is complex and for *Malassezia* a still developing research area.

## *Malassezia* Interactions With Skin and Their Role as a Commensal

Most metagenomic datasets reveal that microbial communities in different skin ecosystems are determined by topography and driven by water (sweat), oil (sebum), or other temporally stable attributes (Findley et al., 2013; Oh et al., 2016). *Malassezia* are enriched in sebaceous zones, particularly breast, back, and head, due to the abundance of the lipid nutrient source (Findley et al., 2013; Jo et al., 2017). *Malassezia* density is associated with the maintenance of skin health (Ashbee and Evans, 2002; Prohic et al., 2016), and *Malassezia* are the most abundant fungi identified at eleven core body sites, all except those on the foot. *M. restricta* and *M. globosa* are by far the most abundant on human skin, with other species occurring at much lower frequency (Findley et al., 2013).

In addition to body site, age plays a role in shaping the skin mycobiome. During gestation, the fetus is exposed to microbes from the placenta, fetal membranes, amniotic fluid, and umbilical cord (Pelzer et al., 2017). Immediately after birth, early skin colonization is influenced by vernix caseosa, a multi-component defense system (anti-microbial sebum) composed of cellular contents, water, lipids, and proteins produced by fetal sebaceous glands during the third trimester (Pickens et al., 2000; Tollin et al., 2005; Michalski et al., 2017; Szabo et al., 2017). Neonates born through vaginal delivery acquire microbial communities from the birth canal and vagina, resembling their mother’s vaginal microbiota, where neonates born *via* cesarean section have skin microbial communities similar to the mother’s skin surface (Dominguez-Bello et al., 2010; Oh et al., 2010; Aagaard et al., 2014; Dunn et al., 2017; Georgountzou and Papadopoulos, 2017). *Malassezia* colonization occurs immediately after birth, when neonatal sebaceous glands are active, being driven by maternal hormones which cross the placental barrier with a progression over the first few months of life to more closely resemble the adult microbiome assemblage (Ashbee and Evans, 2002; Bernier et al., 2002; Ayhan et al., 2007; Nagata et al., 2012). The succession of mycobiome during birth within individual infants is variable either due to mothers vaginal mycobiome or vertical transmission depending on mode of delivery and environmental impact such as caregivers and other sources (Ashbee and Evans, 2002; Bernier et al., 2002; Ward et al., 2018). In contrast to the mother’s skin *Malassezia* colonization, infant skin only contains two percent relative abundance shown by either sequencing, PCR, or culture-based approaches. Despite the higher abundance in mother’s skin, cesarean compared to vaginally born infants have lower *Malassezia* abundance. The reasons are currently unknown but could be biological, environmentally influenced, or a reported technical bias of Internal Transcribed Spacer (ITS) 2 region amplification (Anna and Bazzicalupoa; Bellemain et al., 2010).

As *Malassezia* are lipid dependent colonizers, their skin abundance would be hypothesized to follow the level of sebaceous gland activity and hence lipid level on skin (Ro and Dawson, 2005). Soon after birth (3-6 months), the sebaceous glands become dormant, and *Malassezia* revert to a low abundance. With the onset of puberty, increased lipid levels in sebaceous regions result in a concomitant increase in *Malassezia* abundance (Ro and Dawson, 2005; Prohic et al., 2016). Comparison of skin fungal communities between healthy children and adults showed that *Malassezia* predominates on adults while in children (age < 14) *Malassezia* were present but at lower abundance, and with a more diverse fungal community including Eurotiomycetes and common dermatophytes (Jo et al., 2016). This observation of fungal ecological dynamics may partly be responsible for the prevalence or severity of common skin disorders seen more frequently in children. Together this points to a key protective role of *Malassezia* when fully occupying the skin niche.

## Immune Education, Host Tolerance, and the Response to *Malassezia*

**Figure 2:**
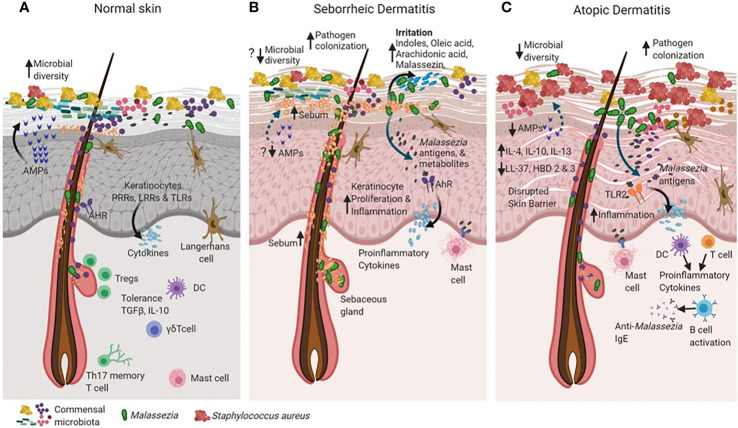
Cross talk between skin and microbiota in healthy and diseased skin states (such as seborrheic dermatitis and atopic dermatitis). The skin and the immune system evolve together with resident microorganisms to establish commensal microbial relationships (for example, *Malassezia* in green). In the healthy state **(A)**, skin maintains high microbial diversity when compared to active disease states in seborrheic dermatitis (SD) and atopic dermatitis (AD) as shown in **(B, C)**. Keratinocytes sense microbial population through recognition of microbial pathogen-associated molecular patterns (PAMPs) motifs *via* their pattern recognition receptors (PRR’s), Leucine rich repeat (LRR’s) containing receptors and Toll-like receptors (TLR’s) as shown in **(A)**. The binding of PAMPs to PRRs, LRRs and TLRs triggers innate immune responses, resulting in the secretion of antimicrobial peptides that can rapidly inactivate a diverse range of pathogenic microorganisms, including fungi, bacteria and parasites. The Langerhans cells interact with microbial antigens in the epidermis to detect barrier breach and maintain homeostasis **(A)**. The skin tolerance is dependent on regulatory T cells, a subset of lymphocytes infiltrate skin, concomitant with hair follicle and skin microbial colonization in based on cytokines TGF-Beta and IL-10 **(A)**. In SD, alterations in sebum content creates favorable conditions for expansion of *Malassezia* as the dominant species that may cause the disease **(B)**. Increased *Malassezia* colonization initiates specific utilization of stratum corneum fatty acids that are converted into by-products such as oleic acid, arachidonic acid which irritates and causes inflammation in the skin. Irritants such as indole and Malassezin could increase keratinocyte (KC) proliferation and induce inflammation **(B)**. Accumulation of histamine in the SD lesions is suggestive of mast cell degranulation **(B)**. In AD there is increased pathogen colonization that causes probable decreased commensal microbial diversity and a defective skin barrier**(C)**. *Malasszeia* and other pathogens could stimulate sub epidermal layers and releases antigens which are recognized by TLR2 receptors feedback to DC or T cell population which stimulates immune response The disrupted skin barrier allows microbial entry in to skin which probably increases cytokines IL-4, IL-10 and IL-13 levels. Circulating anti- *Malassezia* IgE has been reported in AD **(C)**. The figure was created using Biorender.com.

The skin is reliant on commensal microbiota to “train” the immune system and develop appropriate tolerance, host defense mechanisms, and immunity against invading pathogens. Dynamic signals from commensals during early development are used by the immune system to provide heterologous defense mechanisms (Naik et al., 2015). These host interactions are skin-specific, and a resident immune cell population is recruited for establishing innate and adaptive responses in the periphery. One of the foremost factors for skin-microbiota coexistence is host immune tolerance, as shown in **Figure 2**. Tolerance is established after birth, maintained throughout life, and is defined as the capability of the host to suppress the active immune response against itself and certain microbes. The microbes in turn also have mechanisms to evade skin antimicrobial defenses and co-exist with skin (Zhang and Gallo, 2016). There is a significant body of literature, reviewed in brief here, regarding the role of the skin microbiome on normal immune development. However, little is known about the specific role of *Malassezia*. This will be an important area for future research.

Fetal immune development begins as early as nine to fifteen weeks with formation and maturation of multiple cell types including B and T lymphocytes (Hayward, 1983). The fetus maintains a high Th2-biased immune system to prevent proinflammatory Th1-type alloimmune responses to maternal tissues (Philbin and Levy, 2009), but acquires the ability to produce IgG and IgM antibodies at 10 weeks gestation with IgG levels increasing till 22 weeks. The neonates undergo extreme physical and physiological changes at birth, with the skin surface experiencing a drastic shift from aqueous and sterile to dry with a high load of microbial antigens (Hoeger and Enzmann, 2002). The high Th2 dependent-IL6 cytokine levels, formed in the prenatal stage, shield against microbial infections, and these antigens are cleared at birth (Georgountzou and Papadopoulos, 2017).

Dynamic signals from commensals during early development are used by the immune system to provide heterologous defense mechanisms. The evolutionary interaction between cutaneous commensal microbiota and the skin immune system involves changing antigen signals to calibrate immunity against pathogens (Naik et al., 2015; Quaresma, 2019). From the time of birth, the skin microbiota develops as a highly diverse and dynamic ecology which undergoes remodeling due to host and environmental factors and aids in immune education (Grice et al., 2009; Kong and Segre, 2012). However, host tolerance is established in the early phases of immunity development immediately after birth. During this period skin regulatory T cells (Tregs) establish immune tolerance to commensal microbes, preserve homeostasis with skin microbiota, and protect against pathogens (Belkaid et al., 2002). In neonates, there is a steep influx of Tregs into skin within the first two weeks, to mediate tolerance specific to the commensal microbiota (Scharschmidt et al., 2015; Ali and Rosenblum, 2017). As *Malassezia* are among the major commensal fungi in neonates, it can be hypothesized that they may also induce and establish specific tolerance pathways in the skin involving Tregs (**Figure 2**).

In healthy skin, *Malassezia* exist as a commensal and benignly interact with keratinocytes and the immune system, as they reside mainly on the outer skin surface and the follicular infundibulum (Sanmiguel and Grice, 2015; Mittermann et al., 2016). *Malassezia* are detected by the host immune system through keratinocytes and various immune cell populations. The *Malassezia* cell wall components β-(1,6)-glucans, glycolipids, and glycoproteins are recognized by proline rich region (PRR) motifs present in Dectin-2 and Macrophage inducible Ca2+-dependent lectin receptor (Mincle) host cell membrane bound CLR (C-type Lectin) receptors in multiple immune cell types (Ishikawa et al., 2013; Dambuza and Brown, 2015; Underhill and Pearlman, 2015; Sparber and Leibundgut-Landmann, 2017). Langerin, a PRR expressed on epidermal Langerhans cells and a subset of dermal DCs, can recognize beta-glucans expressed on the *Malassezia* cell wall (De Jong et al., 2010; Tateno et al., 2010)(de Jong Mol Immunol 2010; Tateno J Biol Chem 2010). However, what roles these (and other PRRs) play in *Malassezia*-induced commensalism, inflammation, and adaptive immunity is currently not well understood.

Host innate immune activity to *Malassezia* has been well documented with various *in vitro* studies in human keratinocytes based on secretion of proinflammatory cytokines, chemokines and AMPs. *M. furfur, M. globosa and M. restricta* induced increase in expression of Toll-like receptor 2 (TLR-2), IL-8, Human beta-defensin 2 (HBD-2), HBD-3 suggests their role in skin protection. (Baroni et al., 2006; Donnarumma et al., 2014; Georgountzou and Papadopoulos, 2017). These cytokines and chemokines recruit immune cells to skin sites with minimal or compromised barrier, such as the follicle infundibulum, where *Malassezia* may be directly exposed to keratinocytes, tissue resident dendritic cells (DCs), macrophages, myeloid cells and γδ-T cells (Sparber and Leibundgut-Landmann, 2017). *Malassezia* can inhibit phagocyte responses to Toll-like receptor (TLR) stimulation and contribute to cutaneous invariant γδT cell homeostasis through specific indole metabolites and AhR receptor signaling in the skin (Kadow et al., 2011; Vlachos et al., 2012). A murine skin infection model indicates *Malassezia* can induce Th17 immunity (IL-23/17 axis). In healthy human skin, *Malassezia* are also known to modulate the inflammatory cytokine response of CCR6^+^ Th17 memory T cells (Sparber et al., 2019). It is not clear how *Malassezia* mediates an immune response for either a commensal or inflammatory state in human skin.

Innate immune activation in skin enhances the adaptive immune response (Holt and Jones, 2000). Generally, adaptive immune responses are stronger in *Malassezia*-associated diseases, but their status during commensalism is less clear. Emerging evidence also indicates that innate lymphoid cells (ILCs) respond directly to skin fungal populations by producing IL-17 cytokine (Gladiator and Leibundgut-Landmann, 2013; Gladiator et al., 2013). To substantiate the immune response, *Malassezia-*specific immunoglobulins IgG, IgM, IgE, and IgA have also been identified in healthy human sweat and shown to coat *Malassezia* on the skin surface (Page and Remington, 1967; Forstrom et al., 1975; Metze et al., 1991; Cunningham et al., 1992). In summary, although there are studies beginning to describe the immunological response to *Malassezia* as commensals, further studies are required to elucidate precise mechanisms.

## *Malassezia* and Skin Disease

*Malassezia* have now been associated with numerous skin diseases (Gaitanis et al., 2013; Harada et al., 2015; Prohic et al., 2016; Limon et al., 2017; Saunte et al., 2020). These skin conditions are either caused, or exacerbated by, alterations by *Malassezia* in a changing skin environment. *Malassezia* normally exist as a skin commensal without inflicting disease, suggestive of contextual pathogenesis. One possible mechanism of *Malassezia* mediated skin disease is host genetic susceptibility (Deangelis et al., 2005). Skin barrier defects, for example, may change microbiota composition and/or behavior, leading to a corresponding immune inflammatory response. There are two modes by which *Malassezia* interact with the skin. One is direct, where specific *Malassezia* metabolites introduce physiological changes such as irritation. The second is indirect, where immune or allergic pathways are activated and manifest as inflammation (Grice and Dawson, 2017). One emergent challenge is to decipher the role of *Malassezia* as a cause or consequence in its multifaceted interaction with the skin. *Malassezia* can cause hypo- or hyper-pigmented non-inflammatory lesions through interaction with melanocytes, and with mild barrier defects can cause pityriasis versicolor, a common skin infection. Often *Malassezia* metabolites trigger a scalp inflammatory response causing dandruff and in severe situations seborrheic dermatitis, and can invade and inflame hair follicles to cause folliculitis (Theelen et al., 2018). In other inflammatory skin conditions, such as atopic dermatitis and psoriasis, there is increasing evidence about the role of *Malassezia*. As host susceptibility is often a prerequisite for fungal pathogenicity, it remains important for mechanistic investigational studies to be performed longitudinally in susceptible individuals, as important pathogenic mechanisms may well be present in non-susceptible individuals and confound parallel group studies by not inducing the disease phenotype.

### *Malassezia* and Childhood Skin Infections

New-born fungal infections are usually topical with mild symptoms. However, in immune compromised or prematurely born infants’, skin resident *Malassezia* may spread into to blood circulation and disseminate as sepsis, with serious and often lethal consequence. The initial *Malassezia* colonization may cause hypersensitivity reactions in neonatal face, scalp, and neck skin with non-follicular papulopustular eruptions referred to as neonatal acne or sebaceous miliria. It is not clear how these eruptions spontaneously resolve, but they usually do so within weeks. Neonatal and infantile seborrheic dermatitis associated with *M. furfur* shows a scaling scalp, ‘cradle cap’ phenotype and is treated by applying ketoconazole shampoo or petrolatum gently on scalp skin or affected area (Wananukul et al., 2005; Zuniga and Nguyen, 2013). Low birth weight infants are also susceptible to *Malassezia* skin infections and these infants are reported to have high fungal load (Speer et al., 1976; Sperling et al., 1988; Ng, 1994; Sohn et al., 2001; Kaufman and Fairchild, 2004). Proposed mechanisms for preterm neonatal skin and systemic infections involves factors such as the developing fragile skin structure having an incomplete barrier, an under-developed immune system, and microbial transmission from caregivers and hospital sources. Other clinical manifestations of infants of premature births are invasive *Malassezia* infections through catheters in neonatal intensive care units (NICU) (Shek et al., 1989; Pedrosa et al., 2018; Chow et al., 2020). At the onset of puberty there is increased activity of sebaceous glands and lipid content predominantly in facial, scalp and trunk skin. This favors the growth of specific lipophilic microbial populations such as *C. acnes* and *Malassezia.* However, it is not clear which biological factors, including changes in skin microbiota, are causative for acne vulgaris during adolescence (Common et al., 2019; Ramasamy et al., 2019).

### A Role for *Malassezia* in Pityriasis Versicolor

Pityriasis versicolor (PV), also called Tinea versicolor, is a mild, chronic, superficial fungal infection and frequently occurs in children and adolescents when sebaceous activity is maximum. The lesions appear as hyper- or hypo-pigmented (discolored patches), mostly around the trunk and shoulders. Common clinical presentations are mild itch and scaling in affected areas (Gordon, 1951; Gaitanis et al., 2013). This mild cosmetic disease is usually more active in hot and humid weather conditions than temperate climates. *Malassezia* are known to cause PV (Gupta et al., 2002; Crespo-Erchiga and Florencio, 2006). To date *M. furfur*, *M. globosa* and *M. sympodialis* are the most commonly identified species in PV (Saadatzadeh et al., 2001), but due to the numerous changes in *Malassezia* nomenclature and the recent identification of many new species it remains unclear if any specific species is causal for PV. *Malassezia* usually exist as individual spherical yeast, however in PV they become mycelial with profuse hyphal growth and abnormal expansion in the affected site. Interestingly, histopathological staining from skin biopsies shows milder signs of skin barrier defects and no sign of inflammation despite the heavy fungal load. It is possible that *Malassezia* take advantage of a compromised skin barrier and the mycelial form can go in search of a nutrient rich skin layer (Saadatzadeh et al., 2001). It is not clear how and why there is minimal inflammation despite the increased mycelial form and fungal load. Humoral specific IgG response toward *M. furfur* antigens has been reported for PV (Silva et al., 1997). Although PV could be treated by specific antifungal treatments there is high risk of relapse at up to 80%, which severely impacts the patients quality of life (Theelen et al., 2018).

### Direct Pathogenesis: *Malassezia* in Seborrheic Dermatitis and Dandruff

Seborrheic dermatitis and Dandruff (D) are skin conditions found in sebaceous areas with hair. Dandruff is restricted to the scalp and involves itchy, flaking skin without visible inflammation, and is considered a mild non-inflammatory form of SD (Priestley and Savin, 1976; Danby et al., 1993; Warner et al., 2001). SD is a common chronic relapsing inflammatory skin disorder characterized by greasy scales with erythematous skin and exofoliative scaling (oily-yellow desquamation) on the scalp, which may extend to face, ears and upper chest associated with pruritus (Borda and Wikramanayake, 2015). Triggering factors include stress and cold, dry weather (Gary, 2013; Borda and Wikramanayake, 2015). The prevalence is higher in men than women, potentially due to hormonal influence by androgens (Islamoglu, 2019). However, this may also be a result of differences in grooming practices between genders. *Malassezia* have been identified and correlated to SD and D phenotypes, with *M. globosa, M. restricta, M. dermatis* and *M. furfur* associated with these conditions (Nakabayashi et al., 2000; Gupta et al., 2001; Gemmer et al., 2002; Sugita et al., 2002; Kim, 2009). There are three basic etiologic factors for SD and D; *Malassezia*, sebaceous activity, and individual or host susceptibility (Deangelis et al., 2005; Ro and Dawson, 2005). Intrinsic host factors, such as composition of sebum and defective epidermal barrier, likely have an effect on *Malassezia* activity (density, lipase expression and nutrient utilization, immune stimulatory metabolites) that elicits the host inflammatory response. Metabolites such as oleic acid, arachidonic acid, malassezin, and indole-3-carbaldehyde act as skin irritants and are implicated in keratinocyte proliferation and inflammation as shown in **Figure 2**. The causative role of *Malassezia* in SD and D may be assessed through Koch’s postulates (Koch, 1893). As *Malassezia* are found on all humans, Koch’s first postulate cannot be fulfilled (Mcginley et al., 1975). However, it remains unclear whether there are *Malassezia* strain level differences between healthy and D or SD skin that manifest the disease. It is also not known whether the same *Malassezia* strain(s) that exist as commensals in healthy skin contextually become pathogenic due to unknown host environment and susceptibility factors. However, removal of *Malassezia* using antifungals improves D and SD, while removal of bacteria does not, and removal of both bacteria and fungi provides a similar benefit to the removal of fungi alone (Vanderwyk, 1964; Vanderwyk, 1967; Leyden et al., 1976). Furthermore, reintroduction of resistant “*P. ovale*” (likely *M. globosa*) during application of an antifungal (nystatin) are able to induce D and SD flaking (Gosse, 1969). Finally, a specific *Malassezia* metabolite, oleic acid, induces a D like desquamation when applied to scalp free from *Malassezia* and flaking (in individuals previously determined to be susceptible to D and SD) (Ro and Dawson, 2005). These observations fulfill three of four Koch’s postulates and clearly establish the pathogenic role of *Malassezia* in causing D and SD (Gran et al., 2020). The mechanisms of individual susceptibility to D and SD remains unclear, but host genetics implicate immune response (ACT1, C5, IKBKG/NEMO, STK4) and epidermal differentiation (ZNF750). However, it is still not known how disruption to these genetic factors is related to the clinical presentation of D and SD (Jacobs and Miller, 1972; Evans et al., 1977; Mancini et al., 2008; Abdollahpour et al., 2012; Crequer et al., 2012; Nehme et al., 2012; Boisson et al., 2013; Halacli et al., 2015; Karakadze et al., 2018).

### *Malassezia* in Atopic Dermatitis: Pathogenesis or Mutualism?

Atopic dermatitis (AD) and psoriasis are characterized by chronic skin inflammation due to multiple genetic, immune and environmental factors (Bjerre et al., 2017; Weidinger et al., 2018; Nowicka and Nawrot, 2019; Langan et al., 2020). The majority of AD patients have skin barrier dysfunction either due to mutations in genes such as filaggrin and tight junctions or from a more generalized disruption from the presence of Th2 cytokines (Palmer et al., 2006; Sandilands et al., 2009; Jungersted et al., 2010; Rerknimitr et al., 2017; Drislane and Irvine, 2020). The disruption is similar in both instances with a compromised barrier as demonstrated by increased trans-epidermal water loss, high pH, reduced stratum corneum hydration, and altered microbiota (Yang et al., 2020). Increased percutaneous sensitization from microbial products or allergens then produces a vicious cycle stimulating host immunity with resulting dryness, itching and erythema that often progresses into lesional flares and infections (De Benedetto et al., 2012; Lunjani et al., 2018). The incidence of AD is approximately 15% to 20% of children and up to 10% of adults with an age specific disease pattern (Eichenfield et al., 2014; Nutten, 2015). The skin microbiome is strongly associated in pathogenesis of AD with an over growth of *Staphylococcus aureus* at the infected lesions and a distinct microbial configuration in non-lesional skin including alterations in *Malassezia* species (Leyden et al., 1974; Kong et al., 2012; Kobayashi et al., 2015; Chng et al., 2016).

The role of *Malassezia* in AD is supported by both antifungal treatments reducing the severity of symptoms and that application of *Malassezia* extracts or recombinant *Malassezia* antigens on AD subjects exacerbates the phenotype (Zargari et al., 2001; Johansson et al., 2003; Brodska et al., 2014; Glatz et al., 2015; Prohic et al., 2016) (**Figure 2**). *Malassezia* are frequently isolated from and associated with AD, with *M. globosa* and *M. restricta* found more frequently followed by *M. sympodialis* and *M. furfur* (Sugita et al., 2001; Amaya et al., 2007; Kaga et al., 2011). However, one study reported that AD patients yielded exclusively *M. sympodialis* isolates (Sandstrom Falk et al., 2005; Jagielski et al., 2014). One possible explanation why *Malassezia* may be in lower abundance on AD skin is the reduced lipid content associated with the dry skin phenotype (Pilgram et al., 2001; Chng et al., 2016; Theelen et al., 2018). In AD the susceptibility of host to *Malassezia* infection has also been associated with a cytokine gene polymorphism and clinical outcome (Jain et al., 2017).

*Malassezia* are even more strongly implicated in development and persistence of a specific subset of adult head and neck eczema, proposed to be caused by *Malassezia* allergens (Saunte et al., 2020). This subset of patients responds to oral or topical antifungal therapy and have circulating antibodies to multiple *Malassezia* antigens (Scheynius et al., 2002). While the subgroup and antigens were initially found with *M. sympodialis* antigens (Mala S1-9), antigens to other species have also come to light (Andersson et al., 2003). *Malassezia* antigens MGL 1304 (*M. globosa*), Mala s 8, Mala s 13 (*M. sympodialis*), and Mala r 8 (*M. restricta*) have now been shown to be released through *Malassezia* nanovesicles due to the increased skin pH in AD (Selander et al., 2006; Gehrmann et al., 2011; Kohsaka et al., 2018). The antigenic protein MGL-1304 is involved in histamine release and implicated as a component of the AD pathogenesis (Kohsaka et al., 2018). These antigenic proteins are found in sweat, and can cause sweat allergy and an IgE specific AD skin immune response (Hiragun et al., 2013). It is also known that the IgE sensitization profile to skin commensal *M. sympodialis* and specific allergens from the bacterial pathogen *S. aureus* differ between moderate and severe AD patients (Mittermann et al., 2016). One other AD subject sub-group has a CD4^+^ T cell population specifically reacting to *Malassezia* thioredoxin antigen (Mala s 13). Mala S 13 is a homolog of human thioredoxin, and the CD4^+^ T cells cross react with human thioredoxin, leading to AD skin inflammation. A similar triggering mechanism is attributed to Mala S 11, manganese dependent superoxide dismutase (Vilhelmsson et al., 2007; Balaji et al., 2011).

The disrupted skin barrier can provide a constant source for *Malassezia* and its allergens to enter the skin and interact with TLR2 receptors on DCs and keratinocytes. Specific pro-inflammatory cytokines and *Malassezia* specific IgE antibodies are produced through T cell response and B cell activation. The mast cells and DCs also contribute to skin inflammation sustained by cross reacting auto reactive T cells. In AD patients the increased levels of cytokines IL-4, IL-10 and IL-13 suggest inflammatory response and these cytokines are known to reduce the levels of antimicrobial peptides such as Cathelicidin (LL-37) and Human beta-defensins 2 and 3, as well as and skin barrier proteins filaggrin, loricrin and involucrin produced by keratinocytes (**Figure 2**; (Mcgirt and Beck, 2006; Howell et al., 2007; Kim et al., 2008). The growth of pathogens such as *Staphylococcus aureus* and their biofilms in AD skin is probably favored due to lack of abundance of *Malassezia* and reduction of human defensins (Glatz et al., 2015; Chng et al., 2016). *Malassezia* is also known to inhibit the growth of *S. aureus* biofilms through secretion of specific proteases, suggesting its possible role in healthy and AD skin (Li et al., 2018). *Malassezia* antigens are also responsible for activation of NLRP3 inflammasome in DCs and can trigger production of IL-1β, IL-4, IL-5, IL-13 and IL-18 cytokines *in vitro* (Kistowska et al., 2014). A number of these cytokines contribute to inflammation in AD skin as well as other allergic diseases, however, it is not clear whether *Malassezia* antigens are specifically expressed in AD skin or healthy subjects. Additionally, a specific subset of Th17 memory T cells is elicited to *Malassezia* in AD patients aggravating inflammation directly dependent on IL-17 (Sparber et al., 2019). Taken together, there is mounting evidence that *Malassezia* have a role in AD. The specific *Malassezia* contribution to the AD phenotype is not clear and interactions could occur *via* cell wall, cell membrane or lipid metabolite components that stimulate an immune response. However, what has been shown is that antifungals are effective in reducing AD severity, and this should be further explored (Kolmer et al., 1996; Nikkels and Pierard, 2003).

### *Malassezia* in Psoriasis: Evolving to a Pathogenic Relationship in Susceptible Individuals?

Psoriasis affects skin, nails, and joints and is characterized by epidermal hyperproliferation and hyperkeratinisation (Perera et al., 2012; Hugh and Weinberg, 2018). Psoriasis is a T cell mediated autoimmune disease and primarily the result of a combination of genetic and environmental factors (Barker, 1998). Psoriasis (as with AD) has a strong microbial component that could drive or exacerbate the disease phenotype (Fyhrquist et al., 2019; Hurabielle et al., 2020). Certain *Malassezia* species have been associated with particular subtypes of psoriasis such as *M. japonica* and *M. furfur* with psoriasis vulgaris and *Malassezia* yeasts with guttate and scalp psoriasis (Aydogan et al., 2013; Gomez-Moyano et al., 2014; Honnavar et al., 2015). *Malassezia globosa* is the predominant yeast found in scalp psoriatic lesions, followed by *M. furfur* and *M. sympodialis*, but as these are also the same species found on normal scalp there is little evidence they are causal in pathogenesis. However, serum analysis from psoriatic individuals has indicated antibodies against *Malassezia* and its antigens (Squiquera et al., 1994; Gemmer et al., 2002; Jagielski et al., 2014). In Psoriasis, interleukin 23 (IL‐23)/Th17 immune axis has been identified as a major pathway (Blauvelt, 2008; Girolomoni et al., 2017; Li et al., 2020). *Malassezia* can also induce Th1-related cytokines in peripheral blood mononuclear cells *in vitro* (Kanda et al., 2002; Valli et al., 2010) and induce keratinocyte proliferation and proinflammatory cytokine production which could potentially enhance inflammation (Baroni et al., 2004). Topical and systemic antifungal treatments show marked efficacy of improvement in psoriatic lesions (Rosenberg and Belew, 1982; Farr et al., 1985; Amichai, 2004; Armstrong et al., 2016; Beck et al., 2018). In an epicutaneous psoriasis mouse model involving pre-exposure to *Malassezia* followed by Imiquimod (IMQ) there is induction of skin inflammation in a Th17-dependent manner with a transcriptome similar in profile to human psoriatic lesions. Taken together *Malassezia* is likely an exacerbating factor in psoriasis where antifungal treatment can lead to symptomatic improvement but is not the initiating event (Hurabielle et al., 2020). This necessitates attempting more controlled antifungal treatment strategies in psoriasis (Stehlikova et al., 2019). Further work is required to fully understand the role of *Malassezia* in psoriasis.

The strongest evidence that *Malassezia* can directly cause skin disease remains their role in D and SD, where it is clear they play a causal role (Grice and Dawson, 2017; Theelen et al., 2018). They are also very likely to be the causative agent in several less common disorders, including PV and *Malassezia* folliculitis (Gupta et al., 2004). It still remains less clear as to what specific role *Malassezia* may have in inflammatory skin disease pathogenesis. To fully elucidate the role of *Malassezia* in inflammatory skin disease more longitudinal treatment-based studies are needed to elucidate the protective or pathogenic role in susceptible individuals. It is likely that strain level analysis will also aid in defining the molecular mechanisms by which *Malassezia* contextually interact with our skin and how to strategize fungal targeted therapy toward improving clinical outcomes.

## *Malassezia as* a Protector Against Skin Pathogens: Mutualism?

Skin barrier homeostasis is maintained in part due to multi-kingdom microbial communities’ protective roles against pathogens (Byrd et al., 2018). A number of bacterial species have now been shown to provide a protective and synergistic relationship that maintains skin homeostasis (Nakatsuji et al., 2017). It has been hypothesized that the skin fungal mycobiome, primarily *Malassezia*, can protect human skin through its large biomass and spatial-temporal expansion. *Malassezia* metabolize sebum and produce short chain fatty acids (SCFA), such as azelaic acid, which are known to have dual antibacterial and anti-mycotic properties (Brasch and Christophers, 1993). Moreover, *Malassezia* mediated esterification of medium-chain fatty acids generate ethyl ester derivatives with *in vitro* antimycotic activity (Mayser, 2015). *M. globosa* secreted aspartyl protease 1 (MgSAP1) has been shown *in vitro* to disrupt biofilm formation of *Staphylococcus aureus via* hydrolysis of *S. aureus* protein-A (Li et al., 2018). In AD associated microbiomes, there is significant reduction of *Malassezia* as a genus and specifically *M. globosa* resulting in a potentially decreased protective function which could otherwise be restricting *S. aureus* pathogenicity (Chng et al., 2016).

## Conclusion

*Malassezia* are now known to cause skin and scalp disorders, but there remain numerous gaps in the mechanistic understanding of how body site microenvironments affect multi-kingdom skin microbiota composition and function in both healthy homeostasis and disease states. To reach this end goal of defining the role of *Malassezia* as commensal, pathogen, or mutualist we still need to rationalize differences between detection methodologies. Although there is ongoing, rapid advancement in sequencing technologies and metagenomics analysis, it is not yet clear why there is such a large discrepancy between results obtained with cultures, ITS, and metagenomics. It is imperative that future microbiome studies, including any *Malassezia* mediated skin or systemic disease, needs appropriately controlled, robust, and reproducible detection methods which consider and balance both new, cutting edge sequencing techniques and established, well vetted technologies. Also, *Malassezia* have had multiple confusing changes in nomenclature and continuing expansion of the known species, making tracking of the primary literature challenging and assignment of pre-1998 activities to current strains nearly impossible (Theelen et al., 2018). As recent work has shown divergent function between even closely related species and strains, care must be taken in strain identification and assignment of function (Chng et al., 2016; Li et al., 2018). Additionally, many current studies investigating the relationships between microbe and host are limited to parallel group studies of specific pathways in target diseases, limiting understanding of molecular mechanisms that initiate or exacerbate disease progression. Mode of disease onset, function of the skin microbiota, and their role in healthy homeostasis will need to be investigated by implicit longitudinal study design, with appropriate comparison of severity and treatment stages (Grice and Dawson, 2017). These multiple factors have limited our current understanding of the role of mycobiota in healthy skin homeostasis.

In conclusion, the relationship between *Malassezia* and their human host is complex, varies with body site, age, and host susceptibility, and can be in any given circumstance a commensal, pathogenic, or mutualistic relationship. It is important for future clinical studies to account for intrapersonal anatomical variations in the skin microbiota, individual susceptibility, gender, age, seasonality, and ethnicity. Detailed information should also be included to capture the various stressors and perception of skin health or disease, which may promote endocrine and metabolic host changes within the cutaneous microenvironments. Full analysis of these variations will help to delineate the direct influence of microbial alterations in homeostasis of healthy skin and to develop understanding of solid causal relationships.

An improved understanding of the host-*Malassezia* relationship offers tremendous potential for development of treatments to improve skin health outcomes. There are opportunities to develop mycobiome targeted solutions using prebiotic or post-biotic metabolites with the potential to restore healthy skin microbiome and functional attributes such as barrier, dryness, inflammation, and reverse dysbiosis. While most studies to date describe bacterial interactions, it is crucial for future endeavors to address the mechanistic processes between fungal-fungal, inter-kingdom communities, and microbe-host for skin health and disease (Arvanitis and Mylonakis, 2015; Tipton et al., 2018; Zhang et al., 2018). Although there are now an increasing number of detailed studies that demonstrate the mycobiota’s role in commensal and disease states, a substantial knowledge gap remains in understanding fungal virulence determinants and requires further attention.

## Author Contributions

All authors planned the review, read, and approved the final version. SHVC and RS wrote the initial draft. All authors contributed to the article and approved the submitted version.

## Funding

This work was supported by funding from Agency for Science, Technology and Research (A*STAR) and A*STAR BMRC EDB IAF-PP grants_H17/01/a0/004 “Skin Research Institute of Singapore” and H18/01a0/016 “Asian Skin Microbiome Program”.

## Conflict of Interest

The authors declare that the research was conducted in the absence of any commercial or financial relationships that could be construed as a potential conflict of interest.
